# Validation of myocardial infarction diagnosis in patients with congenital heart disease in Sweden

**DOI:** 10.1186/s12872-020-01737-1

**Published:** 2020-10-23

**Authors:** Maria Fedchenko, Zacharias Mandalenakis, Görel Hultsberg-Olsson, Helena Dellborg, Peter Eriksson, Mikael Dellborg

**Affiliations:** Institute of Medicine, Department of Molecular and Clinical Medicine/Cardiology, Sahlgrenska University Hospital/Östra, Diagnosvägen 11, 416 50 Gothenburg, Sweden

**Keywords:** Myocardial infarction, Congenital heart disease, Validation, Swedish patient register

## Abstract

**Background:**

The population of adults with congenital heart disease (CHD) is growing, and increasingly more patients with CHD reach older ages. Patients with CHD are at an increased risk of myocardial infarction (MI) with increased age. Diagnosing MI in patients with CHD can be challenging in clinical practice owing to a high prevalence of aberrant electrocardiograms, ventricular hypertrophy, and heart failure, among other factors. The National Swedish Patient Register (NPR) is widely used in epidemiological studies; however, MI diagnoses specifically in patients with CHD have never been validated in the NPR.

**Methods:**

We contacted hospitals and medical archive services to request medical records for 249 patients, born during 1970–2012, with both CHD and MI diagnoses and who were randomly selected from the NPR by the Swedish National Board of Health and Welfare. Follow-up was until 2015. We performed a medical chart review to validate the MI diagnoses; we also validated CHD diagnoses to ensure that only patients with confirmed CHD diagnoses were included in the MI validation process.

**Results:**

We received medical records for 96.4% (n = 238/249) of patients for validation of CHD diagnoses. In total, 74.8% (n = 178/238) had a confirmed CHD diagnosis; of these, 70.2% (n = 167) had a fully correct CHD diagnosis in the NPR; a further 4.6% (n = 11) had a CHD diagnosis, but it was misclassified. MI diagnoses were validated in 167 (93.8%) patients with confirmed CHD. Of the patients with confirmed CHD, 88.0% (n = 147/167) had correct MI diagnoses. Patients with non-complex CHD diagnoses had more correct MI diagnoses than patients with complex CHD (91.0%, n = 131 compared with 69.6%, n = 16). The main cause for incorrect MI diagnoses was typographical error, contributing to 50.0% of the incorrect diagnoses.

**Conclusions:**

The validity of MI diagnoses in patients with confirmed CHD in the NPR is high, with nearly 9 of 10 MI diagnoses being correct (88.0%). MI in patients with CHD can safely be studied using the NPR.

## Background

Congenital heart disease (CHD) is the most common congenital anomaly affecting about 1% of all living born children [1, 2]. Today, more than 90% of children born with CHD survive into adulthood [3–5]; the number of geriatric patients with CHD is also increasing [6, 7]. With increasing life expectancy, patients with CHD are also at risk of acquired cardiovascular diseases, such as myocardial infarction (MI) [8–11]. Published data on the prevalence of MI in patients with CHD are still relatively scarce; however, several observational cohort studies and large registry studies have shown an increased risk of MI and coronary artery disease (CAD) in patients with CHD compared with patients who do not have CHD [8, 12–14].

Healthcare data based on large national administrative registers is increasingly used in many observational studies [8, 12, 15–18], making it possible to include large patient populations to study a wide range of outcomes in a time-effective and cost-effective manner. It is therefore important to validate the diagnoses in these registers, to ensure that the studied diagnoses are correct. The Swedish National Patient Register (NPR) is a nationwide register that is widely used for epidemiological studies [19]. A diagnosis of MI has repeatedly been shown to have a high level of validity in the NPR [15, 20]; however, an MI diagnosis specifically in patients with CHD has not yet been validated in the NPR.

Diagnosing MI in patients with CHD can be challenging in clinical practice for several reasons; patients with CHD often show abnormal electrocardiogram (ECG) patterns, either as a consequence of previous surgeries, right or left ventricular hypertrophy, coronary anomalies, arrhythmias, atrioventricular (AV) node displacement such as in AV canal defect, congenitally corrected transposition of the great arteries, and univentricular hearts [21]; in addition, as heart failure is relatively common in patients with CHD [22–24], cardiac troponin (cTN) levels can be chronically increased. Further, patients with CHD often report relatively high levels of pain/discomfort [25]. It is also possible that CHD patients are at an increased risk of type 2 MI due to vulnerability for coronary embolization (e.g. patients with Fontan circulation), and oxygen supply/demand mismatch because of high prevalence of arrhythmia and heart failure [26–30].

Therefore, the aim of this study was to validate the diagnosis of MI in patients with CHD in the NPR. We also validated the diagnosis of CHD for patients with MI, to ensure that only patients with a confirmed CHD diagnosis were included in the MI validation process.

## Methods

### Swedish national patient register and cause of death register

The NPR is a nationwide register administered by the Swedish National Board of Health and Welfare. The NPR was funded in 1964 and has had nationwide coverage since 1987. Since 2001, the NPR includes all diagnoses from hospital outpatient clinics; however, diagnoses made in primary care are not included in the register [19]. It is compulsory for hospitals to report to the NPR. Hence, for every hospital admission or outpatient visit, information including the main and complementary diagnoses, admission dates, and hospital and department types are reported to the NPR [19].

The Cause of Death register is also administered by the Swedish National Board of Health and Welfare and contains all causes of death as well as contributory causes [31].

### Study population

The Swedish National Board of Health and Welfare randomly selected 600 patients, born between 1930 and 2012, from the NPR and/or Cause of Death register who had a diagnosis of congenital heart or vascular conditions, using the following International Classification of Disease (ICD) codes: ICD-8: 746–747, ICD-9: 745–747, ICD-10: Q20–28 and myocardial infarction or angina pectoris (ICD-8: 410; ICD-9: 410–411B; ICD-10: I20–I21). Follow-up of both CHD and MI diagnoses started in 1970 and went on until 2015. As we mainly aimed to validate MI diagnoses in the contemporary ICD era, only 100 of the 600 selected patients had MI/angina diagnoses according to the ICD-8 or ICD-9 versions.

From the data received from the Swedish National Board of Health and Welfare, we identified all patients with a CHD diagnosis (ICD-8: 746–746.99; 747–747.59, ICD-9: 745A–747E and ICD-10: Q20–Q26 except Q26.5 and Q26.6, which are vena portae anomalies). Among patients with a CHD diagnosis, we then identified all patients with an MI diagnosis (ICD-8 and ICD-9: 410; ICD-10: I21).

We validated only MI diagnoses that were primary diagnoses for patients identified in the hospital discharge register. For patients registered with MI in the outpatient register, we included both primary and secondary/complementary diagnoses of MI.

Additional file 1: Figure S1 shows the flow chart of patient selection. In total, 249 patients with the CHD diagnoses above and at least one MI diagnosis were included in the study.

### Data collected for validation process

We contacted the individual hospitals and medical archive services in writing, to request the following information regarding the selected MI admissions: the full medical chart and discharge summary, laboratory test reports, first and last electrocardiogram (ECG) during admission, cardiac ultrasound investigation report, coronary angiogram report, and magnetic resonance imaging (MRI) and computed tomography (CT) data, if performed.

When a patient had several admissions for MI in the register, we validated the most recent MI diagnosis. If the patient had been admitted for angina pectoris, we also requested the medical records for the most recent admission owing to angina pectoris. In cases where the medical records from the most recent MI were missing, we retrieved the records for the next most recent admission for MI, or used information about the requested MI episode from other medical records that we received.

To validate the CHD diagnoses for patients with MI, we requested the medical records for the most recent admission or hospital visit with a CHD diagnosis. We also asked for the CHD operative report and the last cardiac ultrasound and cardiac CT or cardiac MRI report. If several CHD diagnoses were found in the register we retrieved medical records for each of them.

Reminder letters were sent out to hospitals and medical archive services that did not respond to the initial letter, and phone calls were also made to non-responding hospitals and medical archive services.

### Validation process

Four of the authors reviewed the medical notes. In unclear cases, the notes were reviewed by the senior cardiologist and discussed until consensus was reached.

#### Validation of MI diagnoses

For validation of MI, we used the Fourth Universal Definition of Myocardial Infarction (2018) [32], which requires elevated cTN over the 99th percentile with a rising/falling pattern, as well as any of the following: symptoms of myocardial ischemia, new ischemic ECG changes, new Q waves, imaging evidence of new loss of viable myocardium or new regional wall motion abnormality in a pattern consistent with an ischemic etiology or evidence of thrombus formation on coronary angiogram or autopsy.

As our study also included MI diagnoses in the era before the use of cTN, a diagnosis was accepted as correct if stated by the physician in charge and supported by information of symptoms and/or ECG pattern and/or increased biomarkers currently used at the time of diagnosis.

We also accepted MI diagnoses as correct even if the criteria of rising/falling pattern in cTN or other biomarkers was not fulfilled, in cases when it was reasonable not to expect a rising/falling pattern in cTN levels (e.g., patient presented late). In a few cases, we also accepted an MI diagnosis as correct when it was stated in the medical records that the patient had experienced an MI and undergone percutaneous coronary intervention (PCI).

Cases were identified as “correct diagnosis”, “incorrect diagnosis” or “insufficient data in the medical records to validate diagnosis”.

#### Validation of CHD diagnoses

For patients with several diagnoses of CHD in the register, we validated the main diagnosis. If that diagnosis was correct, the patient was classified as having a correct CHD diagnosis, even if there were other diagnoses in the register that were not correct. Confirmed CHD diagnoses but where the CHD diagnosis was not correct were classified as “misclassified CHD diagnosis”.

If we did not receive the requested CHD medical records and did not find any evidence of a CHD diagnosis in the MI admission medical record or other medical records, and assessed that it is unlikely that the patient has a CHD diagnosis, the CHD diagnosis was considered incorrect. Suspected and unconfirmed diagnoses such as “suspected left to right shunt” on echocardiography were considered incorrect diagnoses. Diagnoses of bicuspid aortic valves (BAV) with any degree of aortic stenosis were considered correct when there was a diagnosis of either BAV or aortic stenosis.

We classified the CHD diagnoses into complex and non-complex CHD diagnoses and used a widely used CHD classification system originally published by Botto et al. [33] and further modified by Liu et al. [18, 34]. Complex CHD diagnoses were defined as conotruncal defects and severe non-conotruncal defects (i.e. lesion group 1 and 2). Conotruncal defects included the following diagnoses with corresponding ICD 8, 9 and 10 codes: Common truncus (ICD codes 746.09, 745A, Q200), aortopulmonary septum defect (ICD codes 746.09, 745A, Q214), transposition of great vessels (ICD codes 746.19, 745B, Q203, Q205), double outlet right ventricle (ICD codes 746.19, 745B, Q201), double outlet left ventricle (ICD codes 746.19, 745B, Q202), tetralogy of Fallot (ICD codes 746.29, 745C, Q213). Severe non-conotruncal defects included endocardial cushion defects (ICD codes 746.47, 746.46, 746.43, 745G, Q212), common ventricle (ICD codes 746.37, 745D, Q204), hypoplastic left heart syndrome (ICD codes 746.74, 746H, Q234). In the complex group we also included patients with pulmonary atresia (ICD codes 746.64, 746A, Q220). Non-complex CHD diagnoses were defined as all other CHD diagnoses not included in the complex CHD group (lesion groups 3–6).

### Statistical analyses

R version 3.5.2 was used to perform the statistical analyses (R Foundation for Statistical Computing, Vienna, Austria). Microsoft Excel was used to produce the figures. Categorical data are presented as mean and percentage. Continuous data are presented as mean, standard deviation, and percentage of patients or median and interquartile range (IQR).

## Results

### CHD diagnoses

In total, we requested medical records for 249 patients with a diagnosis of CHD and MI. The CHD diagnosis was validated in 238 patients (for 9 patients we did not receive the medical records and in further 2 patients the medical records were incomplete).

**Fig. 1:**
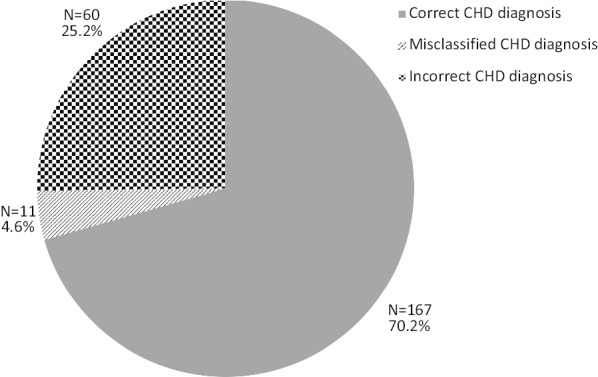
Results of validation of congenital heart disease diagnoses. *CHD* congenital heart disease

Figure 1 shows the results of validation of the CHD diagnoses. In total, 74.8% (n = 178/238) of patients had a confirmed CHD diagnosis. Of these, 70.2% (n = 167) of patients had a correct CHD diagnosis and further 4.6% (n = 11) had confirmed CHD but the CHD diagnosis was misclassified. Half of the patients with confirmed CHD had a diagnosis of atrial septal defect (ASD) or patent foramen ovale (PFO); (50.6%, n = 90), with one-fourth of these PFO (26.7%, n = 24). A total 3.9% (n = 7) of patients had a bicuspid aortic valve as the main CHD diagnosis.

25.2% (n = 60) of patients did not have a CHD diagnosis. The most frequent incorrect CHD diagnosis was VSD (746.39; 745E, Q210), with half of VSD diagnoses being incorrect (50.0%, n = 21). The main reason for this was incorrect assignment of a congenital VSD diagnostic code to patients with post-MI VSD. Also, patients with a valvular disease diagnosis had a high proportion of incorrect diagnoses; of patients with aortic or mitral valvular heart disease diagnoses (including supravalvular aortic stenosis), half (48.5%, n = 16/33) did not have a confirmed congenital lesion.

Slightly more patients with non-complex CHD diagnoses had a correct CHD diagnosis, as compared with patients with complex CHD (71.4%, n = 147 in non-complex CHD compared with 62.5%, n = 20 in complex CHD). Misclassified CHD diagnoses were more common among complex CHD diagnoses. Table 1 shows the distribution of correct, misclassified and incorrect CHD diagnoses grouped according to “non-complex” and “complex” CHD lesion groups.

**Table 1:** CHD diagnoses for complex/non-complex CHD diagnoses and number/percentage of correct/incorrect CHD diagnoses per group

CHD diagnosis	Number of patients	Confirmed CHD		Incorrect CHD diagnosis
		Correct CHD diagnosis	Misclassified CHD diagnosis	
All CHD	N = 238	167 (70.2%)	11 (4.6%)	60 (25.2%)
Complex CHD	N = 32	20 (62.5%)	5 (15.6%)	7 (21.9%)
Non-complex CHD	N = 206	147 (71.4%)	6 (2.9%)	53 (25.2%)

*CHD* congenital heart disease

### MI diagnoses in patients with confirmed CHD

MI diagnosis was validated in 167 patients with confirmed CHD (median age 58.0 (range 0–85) years, 65.3% male); of the medical records requested for 178 patients with confirmed CHD, we received 169 medical records; however, two of these were incomplete. Most validated MI diagnoses were in the contemporary ICD-10 version (81.4%, n = 136).

**Fig. 2:**
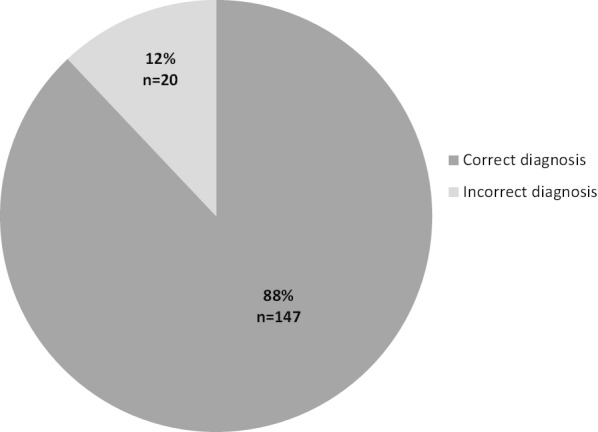
Validation results for myocardial infarction diagnoses in patients with confirmed congenital heart disease

Figure 2 shows the results of MI validation in patients with confirmed CHD. Of the 167 patients with confirmed CHD, 88.0% (n = 147/167) had a correct MI diagnosis. Patients with correct MI diagnoses were older than those with incorrect MI diagnoses; median age 59.0 (range 0–85) years in patients with correct MI diagnosis compared with 46.0 (range 0–75) years in patients with incorrect MI diagnosis.

Twenty patients had incorrect MI diagnoses. The main reason for an incorrect MI diagnosis in the register was typographical error (50.0%, n = 10). In a further two cases, the diagnosis in the medical records did not correspond to the diagnosis in the register. Three patients (15.0%) did not fulfill the diagnostic criteria of MI because of either normal cTN levels or cTN without the timely rise and fall required for a correct diagnosis of MI. Other conditions in which an incorrect MI diagnosis was assigned were pericarditis/perimyocarditis (n = 2), Takutsubo cardiomyopathy (n = 1), unstable angina pectoris (n = 1), and hypokinesia on echocardiography related to a previous surgical procedure that included resection of a part of the myocardium (n = 1).

Slightly more patients with incorrect CHD diagnoses had correct MI diagnoses (93.1%, n = 54/58), in comparison with patients with confirmed CHD diagnoses (88.0%, n = 147/167).

#### Results of MI diagnosis validation in relation to CHD diagnoses

Patients with complex CHD diagnoses had more incorrect MI diagnoses than patients with non-complex CHD. Among patients with complex CHD, only 69.6% (n = 16) had correct MI diagnoses, compared with 91.0% (n = 131) of patients with non-complex CHD. Table 2 shows the distribution of correct and incorrect MI diagnoses in patients with complex and non-complex CHD lesions. Of the 7 patients with complex CHD and incorrect MI diagnosis, 57.1% (n = 4) were due to typographical error.

**Table 2:** All confirmed^1^ CHD diagnoses divided into complex/non-complex CHD lesions, and distribution of correct/incorrect MI diagnoses

CHD	Number of patients	Correct MI diagnosis	Incorrect MI diagnosis
All CHD	N = 167	147 (88.0%)	20 (12.0%)
Complex CHD	N = 23	16 (69.6%)	7 (30.4%)
Type 1 MI		11 (68.8%)	
Type 2 MI		4 (25.0%)	
Other		1 (6.2%)	
Non-complex CHD	N = 144	131 (91.0%)	13 (9.0%)
Type 1 MI		96 (73.3%)	
Type 2 MI		31 (23.7%)	
Other		4 (3.1%)	

*MI* myocardial infarction; *CHD* congenital heart disease

^1^Confirmed CHD includes correct CHD and misclassified CHD diagnoses

More than half of patients (59.2%, n = 87) had a known CHD diagnosis at the time of MI, and 29 patients (19.7%) were diagnosed with CHD while being investigated for a validated MI episode.

#### Clinical characteristics of patients with confirmed CHD and correct MI diagnoses

**Fig. 3:**
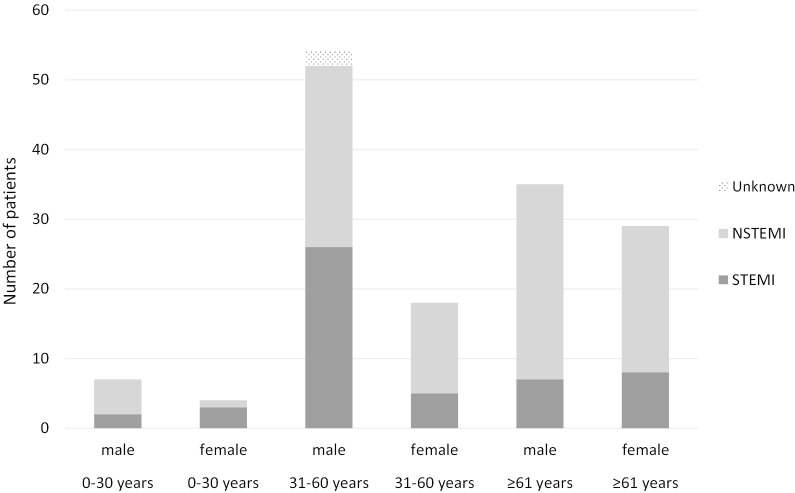
Number of patients with STEMI and NSTEMI according to age decades groups in patients with confirmed congenital heart disease and correct myocardial infarction diagnoses. *STEMI* ST-Elevation Myocardial Infarction; *NSTEMI* Non-ST-Segment Elevation Myocardial Infarction

Table 3 describes the characteristics of patients with confirmed CHD and with correct MI and MI-related information. The median age at MI was 59 (range 0–85) years, and 65.3% (n = 96) of patients were male. 34.7% (n = 51 patients) had ST-elevation MI; information of MI type was missing for 2 patients. The proportion of males and females with STEMI/NSTEMI according to age groups is presented in Fig. 3. STEMI occurred in 31.2% (n = 5/16) of the patients with complex CHD diagnoses, compared with 35.1% (n = 46/131) of patients with non-complex CHD. According to information in the medical records, we assessed that 72.8% (n = 107) of patients had MI type 1 and 23.8% (n = 35) had MI type 2.

**Table 3:** Baseline data and MI related information in patients with confirmed CHD^1^ and correct MI diagnoses

Variable	Total number of patients	Number of patients (%)
Sex	147	
Male		96 (65.3%)
Female		51 (34.7%)
CHD diagnosis	147	
ASD secundum/PFO		73 (49.7%)
VSD		16 (10.9%)
Other		58 (39.5%)
Age at MI	147	59 (IQR 50–67)
Previous MI or ischemic heart disease	147	
Yes		39 (26.5%)
No		107 (72.8%)
Info missing		1 (0.7%)
Symptoms	147	
Typical		118 (80.3%)
Atypical		13 (8.8%)
No symptoms		8 (5.4%)
Info missing		8 (5.4%)
Cardiac enzymes and biomarkers^2^	147	
Lablist available		92 (62.6%)
Values only mentioned in text		40 (27.2%)
Not taken		4 (2.7%)
Info missing		11 (7.5%)
Troponin T or I measured	147	
Yes		95 (64.6%)
No		40 (27.2%)
Info missing		12 (8.2%)
Enzymes/biomarkers elevated	147	
Yes		124 (84.4%)
No		4 (2.7%)
Not taken		4 (2.7%)
Info missing		15 (10.2%)
ECG report available	147	
Yes		82 (55.8%)
No		5 (3.4%)
Mentioned in text		58 (39.5%)
Info missing		2 (1.4%)
ECG	140	
ST elevation		52 (37.1%)
Non-ST elevation (ST-depression, Q-waves, LBBB/RBBB, T-wave inversion)		71 (50.7%)
Other		11 (7.9%)
Normal		6 (4.3%)
CABG	147	
Yes		21 (14.3%)
No		125 (85.0%)
Info missing		1 (0.7%)
Trombolysis	147	
Yes		11 (7.5%)
No		133 (90.5%)
Info missing		3 (2.0%)
PCI	147	
Yes		46 (31.3%)
No		99 (67.3%)
Info missing		2 (1.4%)
Coronary angiogram	147	
Yes		95 (64.6%)
No		49 (33.3%)
Info missing		3 (2.0%)
Coronary angiogram results^3^	95	
Oclusion in 1 vessel		35 (36.8%)
Oclusion in 2 vessels		16 (16.8%)
Oclusion in 3 vessels		22 (23.2%)
No oclusion		16 (16.8%)
Other		5 (5.3%)
Info missing		1 (1.1%)
MI type as stated in medical records	147	
Type 1		2 (1.4%)
Type 2		11 (7.5%)
Other		2 (1.4%)
Info missing		132 (89.8%)
Assessment of MI type	147	
Type 1		107 (72.8%)
Type 2		35 (23.8%)
Type 3		3 (2.0%)
Type 4		0 (0.0%)
Type 5		2 (1.4%)
Known CHD diagnosis before MI	147	
Yes		87 (59.2%)
No		58 (39.5%)
Info missing		2 (1.4%)
CHD diagnosed under investigation for MI	147	
Yes		29 (19.7%)
No		116 (78.9%)
Info missing		2 (1.4%)

^1^Confirmed CHD includes correct CHD and misclassified CHD diagnoses

^2^ TNT/TNI/CK/CK-MB/CK-B/ASAT/ALAT/LD

^3^defined as > 50% stenosis or mentioning in text “significant stenosis” or “occlusion”

Abbreviations: *CHD* congenital heart disease, *ASD* atrial septal defect, *PFO* patent foramen ovale, *VSD* ventricular septal defect, *MI* myocardial infarction, *PCI* percutaneous coronary intervention, *CABG* coronary artery bypass grafting, *ECG* electrocardiogram

#### Cardiovascular risk factors in patients with confirmed CHD and correct MI diagnoses

The most common cardiovascular risk factor in the confirmed CHD population with correct MI was smoking; 25.9% were current smokers (n = 38) and 33.0% (n = 36) smoked previously. Nearly 40% of patients had previously known hypertension (n = 59, 40.1%) and approximately 25% had known hyperlipidemia (n = 36, 24.5%) and diabetes mellitus (n = 35, 23.8%).

## Discussion

In the present study, we found that the validity of MI diagnoses in patients with confirmed CHD (median age 58.0 (range 0–85) years, 65.3% male) was high, with nearly 9 of 10 MI diagnoses being correct (88.0%). The main cause for incorrect diagnosis was typographical errors contributing to 50.0% of the incorrect diagnoses; another common reason was not fulfilling the criteria for a rise and fall in cTN /biomarkers.

Hammar et al. found that 86% of MI diagnoses in the NPR between 1987 and 1995 were fully correct [20]. Another validation study of MI diagnosis in the NPR published in 1993 showed that 95.7% of patients had definitive MI [35]. The results of our study are in line with those of previously published studies that have validated MI diagnoses in the NPR. However, comparisons with our study are difficult to make because both of the abovementioned studies were conducted 20–30 years ago, when the diagnostic criteria for MI was different and cTN levels were not widely used. MI diagnosis in the Swedish NPR has not been validated recently; however, two relatively recent validation studies of MI diagnosis in the Danish Patient Register, published in 2009 and 2003, showed similar trends as in our study, with 81.9% [36] and 93.6% [37].

Patients with CHD represent a rapidly growing patient group owing to the recent advancements in both surgical and medical treatment; patients with CHD are also aging. Compared with patients who do not have CHD, the causes of MI in the population with CHD are multifactorial. Apart from true atherosclerotic CAD, MI in patients with CHD can be caused by emboli, reduced blood supply owing to volume/pressure overload, anomalous coronary arteries, scars or manipulation of the coronary arteries during a procedure, such as the arterial switch procedure in neonates [26, 38–40]. Interestingly, in our study we assessed that 23.8% of the patients had MI type 2 which is higher compared with a Swedish cohort, however, lower compared with international cohorts [41, 42].

There are relatively scarce data on MI in patients with CHD; however, two large registry studies have shown that patients with CHD have an increased risk of MI, as compared with patients who did not have CHD [8, 12]. In our study, we found a high validity of MI diagnoses in patients with confirmed CHD in the NPR. It can be implied from our results that the potentially aberrant ECG pattern and chronically increased cTN values in patients with CHD do not significantly decrease the validity of MI diagnosis.

We found that 74.8% of patients with a CHD diagnosis in NPR had confirmed CHD. A significant proportion of the false CHD diagnoses can be attributed to wrongly classifying post-MI VSDs as congenital VSDs. In addition, patients with valvular heart disease of probable degenerative origin often received a diagnosis of CHD. While administrative databases vary in accuracy of CHD diagnoses [43–46] it seems that in our system, the NPR is dependable for diagnosis of MI in patients with CHD.

Our results of MI and CHD validation are in line with studies that have validated other diagnoses in the NPR. Validation of inflammatory bowel disease (IBD) diagnoses showed a 93% positive predictive value (PPV) for any IBD; however, lower PPV was found for specific diagnoses such as Crohn’s disease (72%) and ulcerative colitis (79%) [47]. Validation studies of ankylosing spondylitis showed a PPV of 70%–89% [48]. A validation study of rheumatoid arthritis in the NPR showed a 91% PPV [49], and pancreatitis showed a 83% PPV [50]. Ludvigsson et al. reviewed studies on validation of diagnoses in the NPR and found a PPV of 85%–95% for most diagnoses [15].

### Strengths and limitations

One strength of our study is the high generalizability, as we validated MI diagnoses from the entire NPR independently of hospital type or geographic area. Another strength is the low number of missing or unavailable medical records. Further, we validated diagnoses in the ICD-8, ICD-9, and ICD-10 versions, allowing us to validate MI diagnoses in different time periods. This is especially important as the MI diagnostic criteria have changed much during recent decades, with the introduction of highly sensitive cTN.

One limitation of this study was that we did not always have access to all clinical data; for example, some ECGs, laboratory blood test results, and data on onset symptoms were missing. It is therefore possible that a small proportion of patients with unstable angina could have been diagnosed with correct MI. In addition, at times the specifically requested CHD medical record was missing and we relied on information in other medical records that were available to us. Although not likely, it is possible that in those cases, the patient actually could have had a CHD diagnosis, although we did not find any evidence of this in the medical records. Further, we classified uncertain/unconfirmed CHD diagnoses as incorrect, to ensure that we validated MI-only patients with confirmed CHD. It is possible that a few patients who we classified as incorrect CHD might have had a confirmed CHD, if further investigation were undertaken (e.g., patients with a suspected shunt on atrial level that was not further confirmed). Further, as the NPR does not include diagnoses from primary care and outpatient clinics before 2001, it is possible that a few diagnoses of non-complex CHD are not included in the NPR.

## Conclusion

Our findings showed that 74.8% of patients with at least one CHD diagnosis had confirmed CHD. Among patients who had a confirmed CHD diagnosis (65.3% male, median age 58.0 (range 0–85)) the validity of an MI diagnosis was high, with nearly 9 of 10 MI diagnoses being correct (88.0%). The main cause for false MI diagnosis was typographical errors, which contributed to half of the false diagnoses. MI in patients with CHD can safely be studied using the NPR.

## Supplementary information

**Additional file 1**. ** Figure S1**: Flow chart of patient selection.

## Data Availability

The data generated and/or analyzed in the current study will not be available to the public due to patient confidentiality and risk of patient identification due to small numbers of patients and rare diagnoses. De-identified data can be made available to other researchers from the corresponding author upon reasonable request.

## Abbreviations

NPR: National Patient Register
CHD: Congenital heart disease
MI: Myocardial infarction
CAD: Coronary artery disease
ASD: Atrial septal defect
PFO: Patent foramen ovale
VSD: Ventricular septal defect
BAV: Bicuspid aortic valve
ICD: International Classification of Disease
IQR: Interquartile range
ECG: Electrocardiogram
cTN: Cardiac troponin
CABG: Coronary artery bypass grafting
PCI: Percutaneous coronary intervention
MRI: Magnetic resonance imaging
CT: Computed tomography
PPV: Positive predictive value
IBD: Inflammatory bowel disease
STEMI: ST-elevation myocardial infarction
NSTEMI: Non-ST-segment elevation myocardial infarction
