# Supplementary material for: Validation of myocardial infarction diagnosis in patients with congenital heart disease in Sweden
Source: BMC Cardiovasc Disord. 2020 Oct 23;20:460. doi: 10.1186/s12872-020-01737-1 (PMC7584083; doi:10.1186/s12872-020-01737-1)
Supplement: Supplementary file 1 — Additional file 1. Figure S1: Flow chart of patient selection. [file 12872_2020_1737_MOESM1_ESM.pdf]

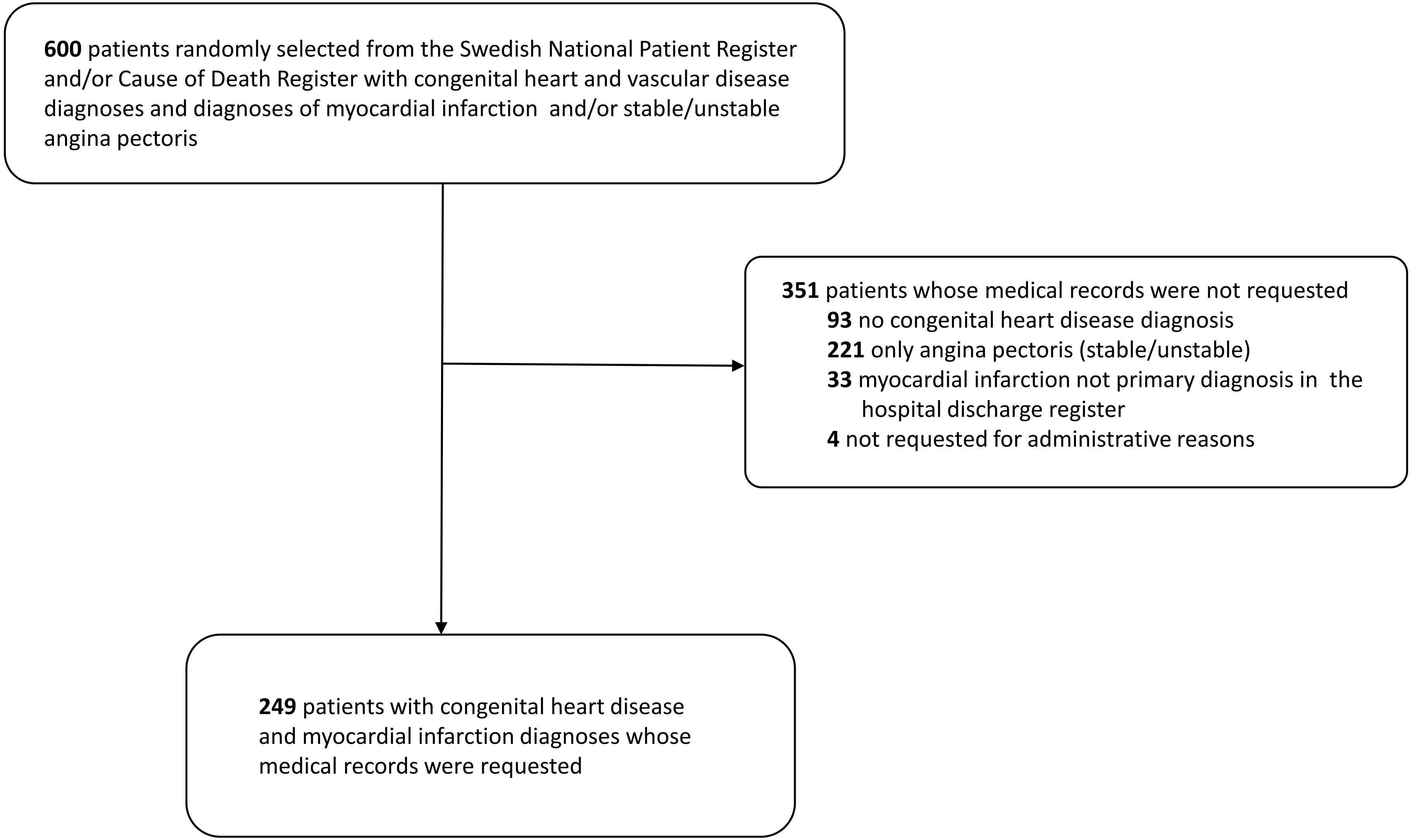
600 patients randomly selected from the Swedish National Patient Register and/or Cause of Death Register with congenital heart and vascular disease diagnoses and diagnoses of myocardial infarction and/or stable/unstable angina pectoris
351 patients whose medical records were not requested
93 no congenital heart disease diagnosis
221 only angina pectoris (stable/unstable)
33 myocardial infarction not primary diagnosis in the hospital discharge register
4 not requested for administrative reasons
249 patients with congenital heart disease and myocardial infarction diagnoses whose medical records were requested
